# Occult Hepatitis B virus infection Among blood donors in Colombia

**DOI:** 10.1186/s12985-014-0206-z

**Published:** 2014-11-29

**Authors:** Wilson Alfredo Rios-Ocampo, Fabián Cortes-Mancera, Juan Camilo Olarte, Angela Soto, Maria-Cristina Navas

**Affiliations:** Grupo de Gastrohepatologia, Facultad de Medicina, Universidad de Antioquia, UdeA. Calle 70 No. 52-21, Medellín, Colombia; Grupo de Investigación e Innovación Biomédica GI2B, Facultad de Ciencias, Exactas y Aplicadas, Instituto Tecnologico Metropolitano (ITM), Medellin, Colombia; Banco de sangre, Cruz Roja Colombiana, Seccional Antioquia, Cra. 52 No. 25-310, Medellin, Colombia

**Keywords:** Hepatitis B virus, Occult infection, Blood donors, HBsAg, Anti-HBc, Genotype F

## Abstract

**Background:**

Hepatitis B virus (HBV) surface antigen (HBsAg) screening in blood banks reduced the risk of HBV transmission through transfusion. However, the detection of occult HBV infection among blood donors is imperative for improving blood safety. The aim of this study was to determine the frequency of occult hepatitis B virus infection among blood donors in Medellin, North West Colombia and to characterize the viral genotypes and mutations.

**Methods:**

Serum samples from blood donors with the serological profile HBsAg-/Anti-HBc+ were evaluated by nested or hemi-nested PCR for HBV genome ORF C, ORF S and ORF X. A pairwise analysis was carried out with deduced amino acids sequence of overlapping S/P region.

**Results:**

A total of 302 serum samples HBsAg-/Anti-HBc+ from donors recruited in a blood bank in Medellin were evaluated by PCR for the HBV genome. Six samples (1.98%) were identified as occult HBV infection. The cases were confirmed by sequencing and viral load analysis. All HBV strains were genotype F, subgenotype F3. The amino acid substitutions sY100H, sV184A, and sK141N were detected in ORF S and rtL108P, rtR110G, rtL180M, rtR192C, rtT150S, and rtL187V in ORF P.

**Conclusions:**

This is the first report and characterization of OBI cases in blood donors in Colombia. Six from 302 donors HBsAg-/Anti-HBc+ were identified. The mutations rtL108P, rtR110G, rtR192C, rtT150S and rtI187V were characterized for the first time in these samples. Further studies are necessary to explore if these mutations could potentially impair HBsAg production.

## Background

In the last three decades a new type of HBV infection has been recognized, Occult HBV infection (OBI). This clinical entity is diagnoses by detection of the HBV genome (HBV-DNA) in liver tissue and/or serum samples, in the absence of detectable hepatitis B virus surface antigen (HBsAg). Additionally, OBI is characterized by a very low viral load (<200 IU/mL) and detection of the antibodies anti-Core (anti-HBc) in 80% of the cases. Moreover, in 20% of the cases there is no evidence of any HBV serological marker [1].

OBI is recognized as a disease with important clinical implications, including the risk of HBV transmission by blood transfusion and organ transplantation and progression to cirrhosis and/or Hepatocellular carcinoma (HCC) [2]. It is proposed that the development of OBI may result from a multifactorial process involving viral and host factors. The hypotheses of OBI pathogenesis include mutations in viral genome, negative regulation of replication by epigenetic mechanisms and/or coinfection with hepatitis C virus (HCV) or Human Immunodeficiency Virus (HIV) [3].

Transfusion-transmitted HBV infection was demonstrated early in 1978, through blood components from HBsAg negative donors, in developing countries where the prevalence of HBV is higher and the procedures for blood donor selection and screening for infection markers are limited [4]. In developed countries nucleic acid amplification testing (NAT) has been introduced as important serum testing for HBV infection in order to improve blood safety [5-7].

The prevalence of OBI found in different studies range widely from less than 1% to as high as 90%. The prevalence rates might be accounted by differences in study populations and techniques for detection of the HBV genome [3,8].

In Colombia, there are few reports of OBI. In a cohort of 50 HIV-1 patients, 12 (8.3%) individuals were classified with a serological profile HBsAg-/anti-HBc+; in another study the frequency of OBI reported was 1% in 103 HIV-1 patients [9,10]. On the other side, Beltran et al analyzed 129 HBsAg-/anti-HBc+ samples obtained from Colombian blood donors in serum pools using NAT; the frequency of OBI reported in this study was 0% [11].

The aim of this study was to determine the OBI frequency and to characterize the viral genotypes and mutations in samples from blood donors with serological profile HBsAg-/anti-HBc+ attending a reference blood bank in Medellin city.

This study provides new information regarding the OBI in blood donors in Colombia and data of HBV strains molecular characterization circulating in the study population.

## Results

### Study population

In a reference blood bank in Medellin city, 14.345 altruistic blood donations were received between February and September 2011 through campaigns conducted in people living in Antioquia state, North West Colombia. The 34.5% of blood donations correspond to first time blood donor.

A total of 29/14.345 cases of HBV infection (0.2%) were identified by detection of HBsAg; however, 310/14.345 (2.16%) blood donors showed a serologic profile HBsAg-/anti-HBc+ (Table 1). According to the technical norms for procedures in blood centers in Colombia, all blood units positive for any serological marker are discarded.

**Table 1:** **Serological profile of donors attending a reference Blood Bank in Medellin, during the period February to September 2011**

			**Serological profile**	
**Month**	**Blood donors**	**First time blood donors (%)**	**HBsAg+**	**HBsAg-/anti-HBc+ (%)**
February	1819	452 (24.8)	4 (0.22)	30 (1.65)
March	1984	922 (46.5)	4 (0.20)	25 (1.26)
April	1744	635 (36.4)	4 (0.23)	61 (3.5)
May	1815	691 (38.1)	1 (0.05)	12 (0.66)
June	1778	589 (33.1)	2 (0.11)	40 (2.25)
July	1716	519 (30.2)	4 (0.23)	61 (3.55)
August	1894	590 (30.9)	6 (0.32)	73 (3.85)
Septiember	1595	552 (34.6)	4 (0.25)	8 (0.5)
Total	14345	4950 (34.5)	29 (0.20)	310 (2.16%)

Serum samples from 302 blood donors HBsAg-/anti-HBc+ were included in this study. Data of Alanine Aminotransferase and bilirubin levels in the OBI cases was not available. None of the 302 samples was positive for anti-HCV or anti-HIV-1 or 2. The mean age of the study population was 39.7 ± 10.97 years; 56% of the subjects were male and 44% female. The information of ethnicity is not included in the interview for donor selection.

### HBV DNA amplification

**Figure 1:**
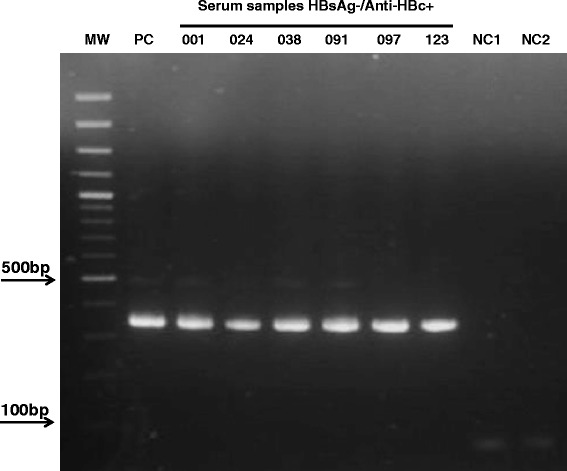
**HBV ORF S detection in serum samples from blood donors with serologic profile HBsAg-/anti-HBc+.** Amplification of small region ORS S (nt 422 to nt 758). MW: molecular weight marker, PC: Positive control serum sample (viral load 50000 IU/mL), NC: negative control PCR 1st and 2nd round, bp: base pairs.

Six samples from 302 were positive for HBV DNA (ORF S) (Figure 1). In these cases, it was possible to amplify a region of 336 nucleotides (nt 422 to nt 758), corresponding to small region of the HBsAg.

These serum samples were subsequently evaluated by hemi-nested PCR of HBV Core region (promoter and ORF Core) and ORF X. One from 6 samples was also positive for HBV DNA ORF X. The ORF Core region was not successfully amplified in any sample (data not shown). The viral load of the 5 serum samples ranged between 18.4 and 224.3 IU/mL. In one case, the viral load was >4.000 IU/mL

Regarding the serological marker anti-HBs, the six samples identified as OBI were positive for anti-HBs. According to the results, 3 donors had anti-HBs titers ≥110 mUI/ml and 3 donors ≤110 mIU/ml (Table 2). The data of HBV vaccination was not available.

**Table 2:** **Molecular and serological characteristics of OBI cases in blood donors attending a reference Blood bank in Medellin, Colombia**

		**Serological profile**		**Virological characteristics**		
**Code**	**HBsAg**	**anti-HBc**	**anti-HBs (mIU/mL)**	**Viral load (IU/mL)**	**Genotype**	**Subgenotype**
001	Negative	Positive	105.6	224.3	F	F3
024	Negative	Positive	108.9	25.9	F	F3
038	Negative	Positive	24.5	18.4	F	F3
091	Negative	Positive	291.4	55	F	F3
097	Negative	Positive	672.7	140.3	F	F3
123	Negative	Positive	>1000.0	4638	F	F3

### HBV genotypes

**Figure 2:**
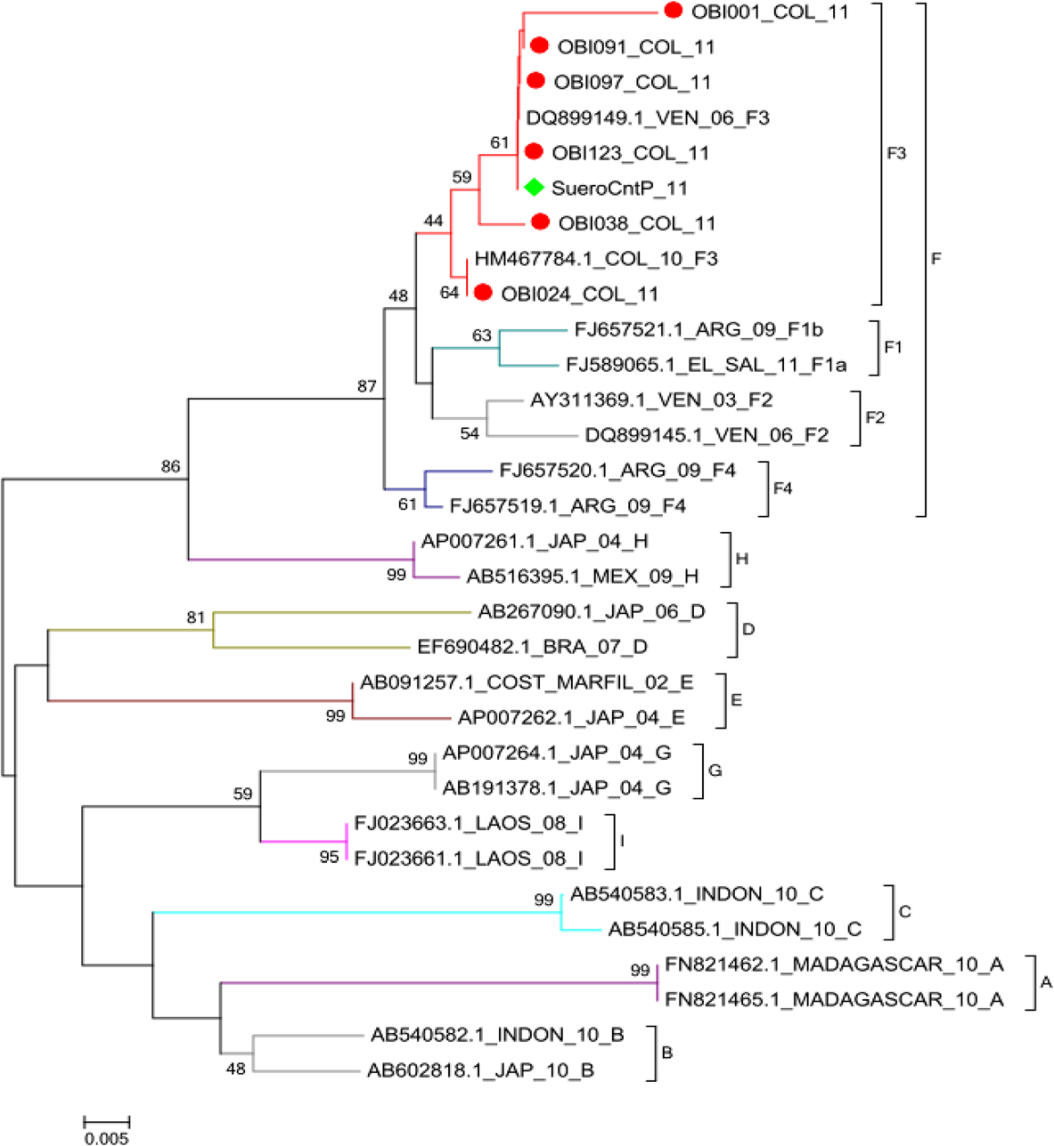
**HBV ORF S phylogenetic tree without root.** For the analysis, it was used a fragment of 267 nucleotides, corresponding to small region of ORFS (nt 449 to nt 716). The sequences of the OBI cases (indicated by red dots) were compared with representative sequences of all HBV genotypes (A to I). Tree was generated by MEGA 5.0 program using Neighbor Joining method. Bootstrap values (indicated with Arabic numerals) were obtained from 1000 replicates, values above 50 are indicated. The green diamond indicates the positive control.

Phylogenetic analyses were conducted by aligning the 6 sequences identified in this study with sequences of HBV genotypes (A to I) available in GenBank (Table 3). After editing of the sequences, it was considered for each one an informative fragment of 267 nt, corresponding to small region of the HBsAg (nt 449 to nt 716). The 6 isolates were grouped in genotype F clade, subgenotype F3 using the NJ method. The bootstrap values for the genotype and cluster branching were obtained from 1000 replicates (Figure 2). Congruent topologies were found between the phylogenetic trees obtained by the ML, MP and, MrBayes methods (data not shown).

**Table 3:** **Sequences of HBV including in phylogenetic analysis**

**GenBank Code**	**Year**	**Country**	**Genotype**	**Subgenotype**
DQ899149	2006	Venezuela	F	F3
HM467784	2010	Colombia	F	F3
FJ657521	2009	Argentina	F	F1b
FJ589065	2008	El Salvador	F	F1a
AY311369	2003	Venezuela	F	F2
DQ899145	2006	Venezuela	F	F2
FJ657520	2009	Argentina	F	F4
FJ657519	2009	Argentina	F	F4
FN821462	2010	Madagascar	A	-
AB602818	2005	Japan	B	-
AB540582	2010	Indonesia	B	-
AB540583	2010	Indonesia	C	-
AB540585	2010	Indonesia	C	-
AB267090	2006	Japan	D	-
EF690482	2007	Brazil	D	-
AB091257	2002	Costa de Marfil	E	-
AP007262	2004	Japan	E	-
AP007264	2004	Japan	G	-
AB191378	2004	Japan	G	-
AP007261	2004	Japan	H	-
AB516395	2009	Mexico	H	-
FJ023663	2008	Laos	I	-
FJ023661	2008	Laos	I	-

### Mutations in the overlapping ORF S/P

The 6 sequences of HBV isolates were aligned and compared with the sequences EF397971, EF397973, FJ589066, DQ899149 and AB036905 genotype F, subgenotype F3 reported in GenBank (Table 3) to identify mutations in ORF S and ORF P [12]. Synonymous and non-synonymous mutations were identified in the ORF S of 4 OBI isolates (Table 4). Regarding the ORF P, it was possible to identify nonsynonymous mutations in the sequence coding for the RT domain, three of them were identified in the same sample (Table 4).

**Table 4:** **Mutations in ORF S and ORF P in OBI cases from blood donors**

**Code**	**ORF S**		**ORF P**
	Synonymous mutations*	Non synonymous mutations*	Non synonymous mutations*
001	A1392G, C1602A	T1387C	T572C, A577G, C787A
024	C1638T	T1640C	C823T
038		A1512T	A697T
091	A1623G		A808G
097			
123			

A. Adenine; G. Guanine; T. Thymine; C. Cytosine.

*Mutations regarding to sequences EF397971, EF397973, FJ589066, AY311370, DQ899149, AB036905 reported in GenBank.

**Figure 3:**
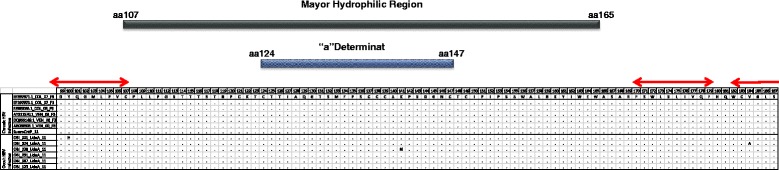
**HBsAg deduced amino acid (aa) alignment.** Comparison of aa substitutions in the Major Hydrophilic Region (MHR) of the HBsAg (aa 99-187), between sequences from OBI samples 001, 024, 038, 091, 097 and 123 and sequences from chronic HBV carriers subgenotype F3 from Colombia and Venezuela reported in GenBank. The aa were presented in single letter code. The lines indicate the regions of the sequences not included in the analysis. SueroCntP 11 corresponds to the positive control. At the top of the alignment, it is represented the MHR by rectangles between aa 107-165 of the HBsAg and the "a" determinant (aa124 to aa147). Red arrows indicate epitopes recognized by cytotoxic T lymphocytes.

**Figure 4:**
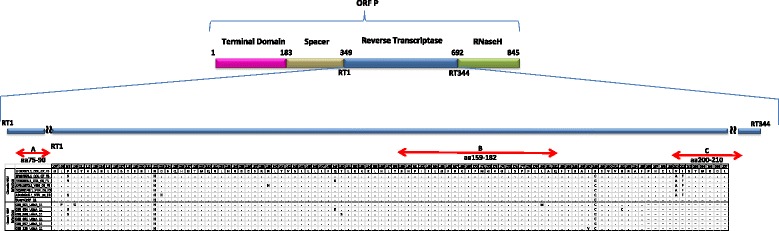
**HBV polymerase deduced amino acid (aa) alignment.** Comparison of aa sequences of OBI samples with chronic HBV consensus sequence subgenotype F3 reported in GenBank. Substitutions were identified on RT polymerase domain (aa rt107-rt195). The aa were presented in single letter code. The lines indicate the regions of the sequences not included in the analysis. SueroCntP 11 corresponds to the positive control. At the top of the alignment, it is represented by rectangles the ORF P and their domains between aa 1 to 845. Red arrows indicate the A to G motives describe in the HBV polymerase.

The deduced amino acid sequences were also analyzed. In the coding region of HBsAg, three amino acid substitutions were identified, sY100H, sV184A and sK141N in samples 001, 024 and 038, respectively (Figure 3); these changes correspond to the mutations T1387C, T1640C and A1512T (Table 4). In the RT domain of the polymerase we identified three amino acid substitutions in sample 001 (rtL108P, rtR110G and rtL180M). Additionally, rtR192C, rtI187V and rtT150S substitutions were observed in samples 024, 038 and 123, respectively (Figure 4).

## Discussion

Colombia has a heterogeneous prevalence pattern of Hepatitis B virus infection, including regions of high, moderate and low prevalence. According to the Colombian National Health Institute, 2.366 cases of HBV infection was reported in 2013 corresponding to an average incidence of 4.67/100.000 inhabitants while the incidence in Antioquia state was 6.07/100.000 for the same period [13].

Antioquia state is one of the most important regions in Colombia due to area (62.840 kms^2^), inhabitants (5.988.458) and industrial development [14]. Medellin, capital of this state, is the second most important city of the country.

The Colombia Health Minister and the National Institute of Health implemented in 1993 the laboratory screening of blood donations, including the serological marker of HBV infection, HBsAg. Until 2012, HBsAg was the only required test for HBV infection; after that it was introduced another marker of HBV infection, antiHBc. Since February 2014, the blood banks in Colombia must discard donations reactive for HBsAg and/or anti-HBc [15]. Actually, 36% of blood units collected before 2011 in Colombia were not being screened for anti-HBc and only 8% were analyzed for detection of nucleic acids by NAT in pools of six units per test [11]. One of the blood banks using screening the anti-HBc in donors since 2000 is the reference blood bank of this study.

The lack of anti-HBc in the test for blood donor is an important risk of HBV transmission from occult hepatitis B infection cases. In Colombia, there are no data about OBI prevalence in blood donor except for the report of 0% of OBI cases in 129 HBsAg-/anti-HBc + serum samples analyzed by NAT in serum pools [11].

The present study is the first one identifying and characterizing OBI cases in blood donors in Colombian donors. The criteria for the diagnosis of OBI in the present study were: i) serological profile markers HBsAg-/anti-HBc+, ii) HBV DNA detection by nested or hemi-nested PCR, iii) sequencing of HBV strains, and iv) viral load <200 IU/mL.

Consequently, it was possible to identified 6 (1.98%) cases of OBI from 302 blood donor samples. Although, the sample 123 showed a viral load of 4638 IU/mL, the case was classified as OBI considering the first three criteria; additionally, HBV escape mutants was not identified in this sample.

The frequency 1.98% is quite similar compared with other studies carried out in blood donor from other Latin American countries with similar epidemiological pattern of HBV infection. In a recently study, the prevalence of OBI was determined in a population of 799 blood donors anti-HBc + living at the State of Amazonas, Brazil, a region of high endemicity for HBV infection. Eight samples (2.7%) were classified as OBI in the study population [16]. In a previous study carried out in Recife, Northeastern Brazil, the frequency of OBI was estimated in 2.5% from 120 samples of blood donors anti-HBc + [17]. However, in two studies of blood donors in Porto Alegre and in Campinas, Southern Brazil, the frequency of OBI was 0% and 6%, respectively [18,19]. The results of these studies could be related to the differences of HBV endemicity between Northern and Southern states of Brazil.

Additional data of OBI in Latin American showed 4.3% OBI cases from 258 HBsAg-/anti-HBc+ samples from blood donors in Caracas, Venezuela, and 6.4% OBI cases from 372 blood donors with serological pattern HBsAg-/anti-HBc + in Yucatan, Mexico [20,21].

On the other side, a high frequency of OBI was described in an Amerindian community in Venezuela; this population has exposure to HBV but low frequency of HBsAg (17%) compared with other amerindian comunities in this country. The HBV DNA was detected in 23/70 serum samples from individuals of the Piaroa community HBsAg-/anti-HBc+ or HBsAg-/anti-HBc-. The high frequency of OBI (32.8%) described in this population could be related to immunosuppression due to multiple parasitic and bacterial infections [22].

Other studies of HBV infection carried out in Amerindian population showed lower prevalence of OBI compared to the frequency described in Piaroa community in Venezuela. The prevalence of OBI reported in Nahuas and Huichol communities from Mexico was 14.2% (41/289) [23]. While the prevalence of OBI in 487 individuals from the Inuit community in North American was 9.7% [24].

In another study of HBV infection in Ameridian communities belonging to four ethnic groups in Argentina (Mbyá-guaraníes, Kollas, Wichis and Sagua-Huarpes) 11 cases of OBI was identified from 59/561 amerindians reactive for HBsAg and/or anti-HBc [25].

The frequency of OBI reported in the studies is related to the prevalence of HBV infection in the study population and the sensitivity of HBV DNA assays. In areas with intermediate to low prevalence of HBV infection, it is expected less than 5% of OBI cases in blood donors with serological profile HBsAg-/anti-HBc+; on the other side, it high prevalence regions of HBV infection, the OBI cases is estimated between 5% to 25% among the blood donors HBsAg-/anti-HBc + [26].

All HBV strains from OBI cases in this study belonged to genotype F, subgenotype F3 as indicated by the phylogenetic analysis (Figure 2). This result is consistent with the epidemiological data in Colombia, where the subgenotype F3 is the most common in HBV infected patients and blood donors [12,27,28].

Indeed, the genotype F, subgenotype F3, was the most important HBV genotype described in two studies carried out with samples from blood donors in Colombia. In the first one, the genotype F was identified in 86% of the samples of blood donors in Bogota and Bucaramanga cities, while the genotypes A, D, C, G were identified in 14% of the samples [12]. In the second one study carried out with samples from blood donors of different cities around the country, the genotype F was also the predominant (77%); however, the genotypes A and G were identified in 15% and 7.7% of the samples, respectively [27]. The predominance of HBV genotype F, subgenotype F3 (83.3%), was also described in patients with end-stage hepatic disease attending a hospital in Medellin city [28].

Interestingly, the genotype F, subgenotype F1b, was the only one identified in Amerindian communities living in Amazonas state, south east of Colombia [29].

The circulation of different HBV genotypes could be related to the numerous ethnic groups present in this country. Indeed, the estimated admixture population in Colombia is American ancestry 0.29, European Ancestry 0.6 and African Ancestry 0.11; although, African ancestry is highest in the Pacific and Atlantic regions (particularly on the Pacific) and highest European ancestry in central region. Meanwhile, American ancestry is highest in the south of the country [30].

Three non-synonymous mutations were detected in OBI cases; Mutations in the S region T1387C, T1640C, and A1512T and the amino acid substitutions sY100H, sV184A, and sK141N, respectively. Two of these mutations (sV184A and sY100H) were found outside of the HBsAg Major Hydrophilic Region (MHR) and therefore outside of the “a” determinant (Figure 3).

Amino acid changes at residue 100 of HBsAg are a frequent event in OBI cases. Indeed, the substitution sY100Cysteine (C) have been described in different studies of OBI cases in blood donors [31], in HIV infected patients [32] and in patients undergoing hemodialysis [33]. Additionally, the mutation sY100Serina (S) was detected in samples from OBI cases [34].

As shown, the 100-residue in HBsAg is often variable in cases of OBI. Cysteine (C), serine (S) and histidine (H) are polar amino acids, and thus it is suggested that the change of this three amino acid can have a similar effect on the antigenicity of the HBsAg. However, further studies are necessary to confirm the frequency of those mutations in OBI cases and its consequence on the synthesis level and antigenicity of the HBsAg.

Huang et al, carried out the molecular characterization of HBV from OBI cases in a cohort of blood donors (n = 38.499). The analysis of the ORF S showed a higher frequency of mutations in MHR (aa 110–160) in OBI cases compared to the donor HBsAg + (Chronic infection). Interestingly, the MHR mutation frequency was not correlated with the serum level of HBsAg. Thirteen MHR mutations (G119R, P120T, C124R, C124Y, I126S, Q129R, S136P, C139R, T140I, K141E, D144A, G145A, and G145R) were detected and functionally characterized *in vitro* and *in vivo.* Some of these mutations, including K141E, impaired the secretion of virions and subviral particles and reduced the reactivity of antibodies anti-HBs used in immunoassays [35]. In the present study was also described a mutation at position 141 (sK141N).

Six amino acid substitutions rtL108P, rtR110G, rtL180M, rtR192C, rtT150S and rtI187V were detected in the sequence of 4/6 OBI cases, three of them were present in sample 001. The mutations were found in the reverse transcriptase (RT) domain of the viral polymerase (nucleotides 349 to 692).

The rtL180M substitution has already been reported in the literature. Moreover, the change of Leucine to Methionine has been described in association with other mutations in the YMDD polymerase domain, in particular with the substitutions rtM204V and rtM204I, related to interferon resistance and polymerase protein dysfunction [36,37]. However, in this study it was not possible to analyze the sequence coding the YMDD domain, because the size of the fragment analyzed (only 267 nucleotides) it was no enough. Interestingly, other studies have shown the emergency of mutations in the ORF S that also caused mutations in the ORF P. Lai et al, demonstrated that mutations sL173F, sI195M, and sY200H also related to these mutations rtA181V, rtM204V, and rtV208A, all of them associated to interferon resistance. However, in the present study we did not identified mutations in the ORS S overlapping to the ORF P or viceversa [38].

This is first report of the mutations rtL108P, rtR110G, rtR192C, rtT150S and rtI187V. Further studies are necessary to characterize *in vitro* and *in vivo* these mutations and its effects on antigenicity and on HBV replication.

## Conclusion

The Colombian National Institute of Health is the institution responsible for the blood system and the strategies and policies for safe blood in this country. Recently, it was included the marker anti-HBc in the official regulation for blood screening donations. The anti-HBc is an important serological marker for detection of cases of OBI and for reducing the residual risk of HBV transmission.

In this study for the first time in Colombia, we identify and characterized OBI cases in blood donors by molecular and serological tests. A frequency of 1.98% of OBI cases was found in 302 blood donors HBsAg-/anti-HBc+ attending a reference blood bank in the second most important city of Colombia. Additionally, three nonsynonymous mutations were found in the ORF S, S region, T1387C, T1640C, and A1512T; whereas 6 amino acid substitutions (rtL108P, rtR110G, rtL180M, rtR192C, rtT150S and rtI187V) were found in the RT domain. Some of these mutations could be related to the pathogenesis of OBI.

This data is important for the Colombian Blood system and the epidemiology of HBV infection in Colombia and in Latin America. Further studies of OBI in blood donors from different geographical regions in Colombia are necessary considering the endemic patterns of HBV infection in this country.

## Methods

### Serum samples

This study was performed between February and September 2011, among blood donors from a representative blood bank of Medellin. Units of blood components were individually screened for HBsAg and anti-HBc using commercial Kits (Bioelisa HBsAg 3.0 Biokit and anti-HBc Biokit).

The detection limit of the HBsAg assay is 0.100 units/mL (standards Paul Ehrlich Institute, Germany) and 0.125 IU/mL (International standards from the World Health Organization (WHO ref. 80/549) [39].

A total of 310 samples with the serological profile HBsAg-/anti-HBc+ were identified. Samples were anonymous and identified only by alphanumerical codes, after signing the informed consent provided by the blood bank. The protocol was approved by the ITM ethical committee.

### Detection and quantitation of HBV-DNA

Total DNA was extracted from 200 μL of serum using a commercial kit (QIAamp DNA Blood mini Kit, QIAGEN) according to the manufacturer’s instructions. Three conserved regions of the HBV genome were amplified by nested and heminested PCR (ORF S, ORF C, and ORF X) according to the method described by Zeng et al [40], Schaefer et al [41], Gunther et al [42], and Hu et al [43]. A serum sample obtained from a patient with chronic HBV infection was used as positive control of the assays. All assays were performed in duplicate.

The sensitivity of the PCR reactions for ORF S, ORF C (preCore-Core), and ORF X was determined by serial dilutions of the positive control with Fetal Bovine Serum (FBS). The detection limit of the PCR protocol to amplify the ORF S was 50 IU/mL while for the ORF Core and for the ORF X was 500 IU/mL (data not shown).

The viral load was determined by real-time PCR (SsoFast EvaGreen supermix, BIORAD) using the protocol kindly provided by Dr. Tonya Mixson-Hayden from the Center for Disease Control and Prevention (CDC)-Atlanta, USA. The ORF S amplification by qPCR was performed using the HBV425LR and HBV359F oligonucleotides described by Mixson-Hayden [44].

### Anti-HBs detection

The anti-HBs levels were determined in serum samples using the prototype Microparticle Enzyme Immunoassay (MEIA) and the automated Abbott AxSYM® AUSAB®.

### HBV sequencing and phylogenetic assay

The amplified products were sequenced using the automated DNA sequencer BigDyeTM terminator (Macrogen, Inc. Seoul, Korea). Sequences were evaluated with the BLASTN (Basic Local Alignment Search Tool form the National Center for Biotechnology Information) program and edited using the SeqMan program (DNASTAR). The sequences were then aligned with HBV sequences genotypes (A to I) from GenBank (Table 3); the alignment was performed using BioEdit [45]. Phylogenetic analyses were performed using MEGA 5.0. For genotyping, the best model of nucleotide substitution and the distance matrix with the p-distance model was determined. Phylogenetic inferences were conducted using methods of Maximum Likelihood (ML), Maximum Parsimony (MP), Neighbor Joining (NJ) and MrBayes. For genotype identification we determined the best model of nucleotide substitution which corresponded to the *Kimura 2-parameter + Gamma distributed (G)*. Phylogenetic trees were constructed using the MEGA 5.0 program [46].

### Analysis of amino acid deduced sequences

The amino acid deduced sequences of OBI cases confirmed by sequencing were aligned with Colombian and Venezuelan sequences of chronic HBV infection cases of blood donors subgenotype F3 reported in GenBank (Table 3). Amino acid substitutions in the MHR of the HBsAg (aa 99-187) and RT polymerase domain (aa rt107-rt195) were identified using BioEdit program. Variability between groups was also compared.

### Statistical analyses

Descriptive statistics was applied using the SPSS software (version 14.0). Measures of central tendency as mean and the percentages were included to describe the variables. Genetic diversity between OBI HBV sequences and HBsAg+ reported in GenBank were compared by using Fisher’s exact test.
